# Determination of the quantitative content of chlorophylls in leaves by reflection spectra using the random forest algorithm

**DOI:** 10.18699/VJ21.008

**Published:** 2021-02

**Authors:** E.A. Urbanovich, D.A. Afonnikov, >S.V. Nikolaev

**Affiliations:** Novosibirsk State Technical University, Novosibirsk, Russia; Institute of Cytology and Genetics of Siberian Branch of the Russian Academy of Sciences, Novosibirsk, Russia Novosibirsk State University, Novosibirsk, Russia; Institute of Cytology and Genetics of Siberian Branch of the Russian Academy of Sciences, Novosibirsk, Russia Moscow State Academy of Veterinary Medicine and Biotechnology – MVA named after K.I. Skryabin, Moscow, Russia

**Keywords:** random forest, remote methods, leaf optics, pigments, случайный лес, дистанционные методы, оптика листа растения, пигменты

## Abstract

Determining the quantitative content of chlorophylls in plant leaves by their reflection spectra is an important task both in monitoring the state of natural and industrial phytocenoses, and in laboratory studies of normal and pathological processes during plant growth. The use of machine learning methods for these purposes is promising, since these methods allow inferring the relationships between input and output variables (prediction model), and in order to improve the quality of the prediction, a researcher may modify predictors and selects a set of method parameters. Here, we present the results of the implementation and evaluation of the random forest algorithm for predicting the total concentration of chlorophylls a and b from the reflection spectra of plant leaves in the visible and infrared wavelengths. We used the reflection spectra for 276 leaf samples from 39 plant species obtained from open sources. 181 samples were from the sycamore maple (Acer pseudoplatanus L.). The reflection spectrum represented wavelengths from 400 to 2500 nm with a step of 1 nm. The training set consisted of the 85 % of A. pseudoplatanus L. samples, and the performance was evaluated on the remaining 15 % samples of this species (validation sample). Six models based on the random forest algorithm with different predictors were evaluated. The selection of control parameters was performed by cross-checking on five partitions. For the first model, the intensity of the reflection spectra without any transformation was used. Based on the analysis of this model, the optimal ranges of wavelengths for the remaining five models were selected. The best results were obtained by models that used a two-point estimation of the derivative of the reflection spectrum in the visible wavelength range as input data. We compared one of these models (the two-point estimation of the derivative of the reflection spectrum in the range of 400–800 nm with a step of 1 nm) with the model by other authors (which is based on the functional dependence between two unknown parameters selected by the least squares method and two reflection coefficients, the choice of which is described in the article). The comparison of the results of predictions of the model based on the random forest algorithm with the model of other authors was carried out both on the validation sample of maple and on the sample from other plant species. In the first case, the predictions of the method based on a random forest had a lower estimate of the standard deviation. In the second case, the predictions of this method had a large error for small values of chlorophyll, while the third-party method had acceptable predictions. The article provides the analysis of the results, as well as recommendations for using this machine learning method to assess the quantitative content of chlorophylls in leaves.

## Abstract (ru)

Определение количественного содержания хлорофиллов в листьях растений по их спектрам отражения – важная задача как при мониторинге состояния естественных и промышленных фитоценозов, так и в лабораторных исследованиях нормальных и патологических процессов в ходе роста растения. Применение для этих целей методов машинного обучения является перспективным, поскольку они позволяют «автоматически» строить решающие правила для получения результата (модель предсказания), а исследователю (для повышения качества предсказания) остаются модификация предикторов и выбор множества параметров метода. В статье приведены результаты построения решающих правил алгоритмом случайного леса (random forest) для предсказания суммарной концентрации хлорофиллов a и b по спектрам отражения листьев растений в видимом и инфракрасном (ИК) диапазонах длин волн. Набор данных взят из открытых источников. Они включали 276 образцов листьев 39 видов растений. При этом 181 образец получен при анализе листьев белого клена (Acer pseudoplatanus L.). Спектр отражения представлен в диапазоне 400–2500 нм с шагом 1 нм. Обучение происходило на 85 % образцов A. pseudoplatanus L., оценка качества предсказания – на оставшихся 15 % образцов этого вида (валидационная выборка). Построено шесть моделей на основе алгоритма случайного леса с разными предикторами. Подбор управляющих параметров осуществляли при помощи перекрестной проверки на пяти разбиениях. Предикторами первой модели выступали имеющиеся значения по спектру отражения без какой-либо обработки с нашей стороны. После проведения анализа этой модели были выбраны диапазоны длин волн предикторов для оставшихся пяти моделей. Лучшие предсказания имеют модели с разностной производной спектра отражения в видимом диапазоне длин волн. Модель с первой производной спектра отражения в диапазоне 400–800 нм с шагом 1 нм брали для сравнения с моделью других авторов. Этой моделью выступает функциональная зависимость с двумя неизвестными параметрами, подбираемыми методом наименьших квадратов, и двумя коэффициентами отражения, выбор которых описывается в настоящей статье. Сравнение результатов предсказаний модели с применением алгоритма случайного леса проводили как на валидационной выборке клена, так и на выборке из других видов растений. В первом случае предсказания метода на основе случайного леса имели меньшую оценку среднеквадратического отклонения. Во втором случае предсказания этого метода были с большой ошибкой при малых значениях хлорофилла, в то время как сторонний метод имел приемлемые предсказания. В статье приводятся анализ результатов и рекомендации по применению этого метода машинного обучения для оценки количественного содержания хлорофиллов в листьях.

## Introduction

Pigments are low-molecular-weight compounds that give color to plant organs and play an important role in their life, performing photosynthetic, protective and metabolic functions. In terrestrial plants, the most well-known pigments are chlorophylls (which provide the green color of plant organs and play a crucial role in photosynthesis), carotenoids (which give red and yellow color, also participate in photosynthesis), anthocyanins (which give a purple color, perform protective functions), as well as a number of other compounds (Croft, Chen, 2018). Photosynthetic pigments, chlorophylls and carotenoids, attract the most attention from researchers; they have different absorption spectra and perform different functions in the process of photosynthesis, which is due to structural differences between the molecules of these substances.

Chlorophyll in plants is represented by two types of molecules, a and b, which have structural differences and differ in their light-absorbing properties (Du et al., 1998). It allows photosynthetic organisms to collect sunlight at different wavelengths to maximize the light energy available for photosynthesis. Changes in the concentrations of photosynthetic pigments are closely related to the physiological state of plants. For example, when the leaves of plants wither, there is a rapid decrease in the concentration of chlorophylls compared to carotenoids, thereby increasing the ratio of carotenoids to chlorophylls causes the leaves to turn red and yellow (Croft, Chen, 2018). The content of pigments, in particular chlorophylls a and b, can thus serve as an indicator of the state of plants during normal growth and during the development of infections, as well as stress, photosynthetic activity, metabolic disorders, etc. (Młodzińska, 2009). The need to determine the physiological state of plants often arises in the course of solving many scientific and practical problems, so methods for assessing the content of pigments in plant organs and tissues are constantly being developed and improved.

Quantitative and qualitative information about pigments can be obtained using chemical methods (Lichtenthaler, 1987; Porra et al., 1989; Wellburn, 1994). However, for many tasks, a more convenient approach is to use remote methods based on the light reflection spectra from the plant leaf (Horler et al., 1983; Curran et al., 1990; Gitelson et al., 2001, 2003). The reflectivity of the leaf in the optical and infrared (IR) wavelengths (400–2500 nm) depends on various biochemical and physical factors, including the content of chlorophyll and other leaf pigments, nitrogen, water, as well as on the internal structure of the leaves and the characteristics of their surface (Croft, Chen, 2018). Plant pigments are characterized by the absorption of electromagnetic radiation in the visible (400– 700 nm) and near-IR (1300–2500 nm) wavelength ranges. The absorption of the leaf components in the near-infrared region in the range of 750–1300 nm is low, since in this wavelength range there is an intense reflection from the components of the internal structure of the leaves. Thus, the reflection coefficient in the near-IR range depends on both the concentration of enzymes and the structure of the leaf. All these facts make it possible to use remote observation methods in both the visible and near-infrared wavelength ranges to monitor the physiological state of plants (Merzlyak et al., 2003; Alt et al., 2020).

One of the approaches to estimating the content of chlorophylls from the reflection spectrum is to select empirical dependencies (indices) between the reflection coefficients at certain wavelengths, the choice of which is also an important part of the method, and the content of chlorophylls (Horler et al., 1983; Curran et al., 1990; Gitelson et al., 2001, 2003; Suo et al., 2010; Nikolaev et al., 2018). The success of such a “classical” approach directly depends on the depth of our understanding of the physics of the process.

Currently, machine learning methods are often used to predict the characteristics of biological objects (Doktor et al., 2014; Feng et al., 2020). Their advantage is that usually a complex nonlinear dependence on many variables can be approximated with the necessary accuracy by machine learning methods. In simple cases, the data is fed to the program input without any processing, however, the accuracy of the predicted parameter will be quite high. Each machine learning method has its own ways to improve the accuracy of the prediction, for example, by varying the control actions. There are also ways to transform the input data to improve the result. Thus, in the analysis of spectra, the calculation of the derivative makes it possible to eliminate additive components and highlight such characteristic features of the spectrum as the positions of maxima, minima, and points

The aim of our research was to develop a machine learning method using a random forest algorithm to predict the total concentration of chlorophylls a and b in plant leaves from the values of the reflection spectra in the visible and infrared wavelength ranges. The accuracy of the prediction is evaluated in comparison with the results obtained from the analytical functional dependence, and the advantages and disadvantages of both approaches are determined.

## Materials and methods

**Experimental data.** The characteristics of the leaf reflection spectra at different concentrations of chlorophylls a and b were downloaded from the EcoSIS database (ecosis. org), set angers2003 (Jacquemound et al., 2003; Féret et al., 2008). 276 leaf samples of 39 plant species were examined. 181 samples are the leaves of sycamore maple (Acer pseudoplatanus L.). The data on the reflection spectrum are presented in the range of 400–2500 nm with a step of 1 nm. The ASD FieldSpec spectrum radiometer is used for this purpose; the pigment concentrations were determined by the Lichtenheler method and are presented in units of measurement of µg/cm2 (see details in (Jacquemound et al., 2003; Féret et al., 2008)).

**Mathematical statement of the problem.** Let there be a general set of Rgen λ of all possible reflection coefficients of plant leaves for given wavelengths λ and Chl gen – the values of the sum of the concentration of chlorophylls a and b corresponding to Rgen λ . We have an Rλ – subsample of Rgen λ and Chl – values of the sum of the concentration of chlorophylls a and b corresponding to Rλ. It is required to construct the functional f: Rgen λ → Chl gen from the set (Rλ, Chl ). Moreover, since this idealized functional cannot be implemented, we get an approximating functional: ~ f : Rλ → Chl. 

**Building a prediction model using the random forest method.** The random forest (RF) method was chosen for constructing the functional (Breiman, 2001; Hastie et al., 2009). It allows you to get the accuracy of the prediction of the target function, as a rule, higher than in the case of linear regression methods. The idea of the algorithm is to apply an ensemble of decision trees. Each decision tree in this ensemble sets a piecewise constant function, which is obtained by minimizing the loss function (for example, the mean square of the deviation). The algorithm combines two main ideas: the Breiman bagging method (Breiman, 1996) and the random subspace method proposed by T.K. Ho (1998). In our work, we used the implementation of the random forest method from the sklearn library (scikit-learn.org) of the Python language.

To predict the chlorophyll concentrations by the random forest method, several models that differed in the input data sets were taken. First, each set was characterized by an interval of wavelengths, the intensity of reflection at which was taken into account. In total, several sets of intervals were considered: 400–2450, 400–800 nm, and a combined set of two intervals of 500–600 and 680–740 nm. Second, the models differed in the type of input data. These included the values of the intensity of the reflection spectra at certain wavelengths (base data type), the values of the first derivatives of the spectral curves for the same wavelengths (der data type), and the values of the second derivatives (der2 data type). Some models were based on only one data type, while others shared multiple data types. Such combinations were marked with a summation sign (for example, base+der).

In this paper, six models have been considered. They are designated as RF-(X–Y)-Z, where (X–Y) – intervals of wavelengths, Z – type data model: RF-(400–2450)-base (the intensity of the spectrum in intervals of wavelengths 400–2450 nm); RF(400–800)-base (the intensity of the spectrum in intervals of wavelengths 400–800 nm); RF(400–800)-base+der (intensity spectrum and the first derivative in the intervals of wavelengths 400–800 nm); RF(400–800)-der (first derivative in the intervals of wavelengths 400–800 nm); RF(400–800)- der+der2 (first and second derivatives in the interval of wavelengths 400–800 nm); RF(500–600; 680–740)-base+der+der2 (intensities, first and second derivatives in the wavelength ranges 500–600 and 680–740 nm).

As an approximation of the derivative of the spectral curves, the first-order finite difference with a change equal to 1 was used, which was calculated by the formula Di = Ri – Ri–1. In this calculation, there is no derivative for the first value. For simplicity, the finite difference is referred to the derivative throughout the text. The second derivative was calculated as the derivative of the derivative of the spectral curve.

When configuring the random forest algorithm, the following control parameters were selected:

- max_depth: [2, 3, 4, 5, 6] – the maximum depth of the tree;
- max_features: [2, 7, sqrt, log2, auto] – the number of features that the partition is searched for (auto – all features);
- n_estimators: [5, 10, 15, 30, 40] – the number of trees in the random forest ensemble;
- random_state: 20200605.

The specified parameters of the algorithm were selected by cross-checking on five samples of the same size obtained from a randomly mixed initial training sample. Four subsamples were used for training the model, and the fifth one was used for testing it. To determine the best control parameters, the test results (mean square deviation of the target indicator – mse) were averaged between models with the same control parameters (i. e., obtained during cross-validation) and sorted. The control parameters for which the average mse is the minimum are the best. As the final model, one of the five models with the best control parameters is selected, which has the minimum mse when tested among the models obtained by the cross-validation method

The maximum depth of the trees is chosen to be 6, which gives 2^6^ = 64 intervals for partitioning the parameter space, despite the fact that the sample length taken to build the model

is 123. The depth increasing could lead to overfitting. The number of trees in the forest (up to 40) may seem redundant for 123 sample values, but the parameters of each of the decision trees were selected on different subspaces (since the random subspace method is used), and the dimension of the features was always greater than the number of elements in the sample.

It should be noted that the algorithm implemented in the sklearn library allows us to obtain the informativeness of each of the model features and select the most informative ones for the obtained decision rules (Breiman, 2001; Hastie et al., 2009; Louppe et al., 2013).

**Construction of empirical functional dependencies.** As a functional of ~ f : Rλ → Chl we additionally chose an empirical dependence from the work (Gitelson et al., 2003) (the GGM method, which we named after the authors’ surnames), represented by the expression (Formula (1))

**Formula:**
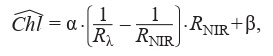
(1)

where Chl is the total concentration of chlorophylls a and b; Rλ is the reflection coefficient at the wavelength λ; RNIR is the reflection coefficient in the near-infrared range (for example, at a wavelength of 800 nm); α and β are selected in such a way as to minimize the selected loss function. A.A. Gitelson and co-authors (2003) recommend choosing wavelengths from the range λ [525; 555] [695; 725]. According to the authors, the advantage of this algorithm is that the RNIR coefficient “corrects” the influence of the plant tissue structure on the reflection spectrum and allows us to extend the found function to plants with different leaf structure. 

**The comparison of methods for predicting the concentration of chlorophyll.** The sycamore maple sample from the angers2003 data set was randomly divided into a training and a validation sample in the ratio of 85 : 15. For the methods used in this work for predicting the random forest algorithm (RF) and functional dependence (GGM), the optimal parameters are selected on the training sample. The quality control of the algorithms is carried out on a validation sample represented by a sycamore maple and on a sample of non-maple samples. The following metrics were used to evaluate the accuracy of predicting chlorophyll concentrations: mse, mean absolute error (mae), and determination coefficient R2. The formulas for calculating metrics are as follows (Formula (2)):

**Formula:**
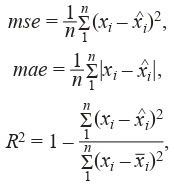
(2)

where x is the true values; ^ x is the predicted values; n is the number of samples, and x is the mathematical expectation for the true values. In terms of optimization, mae and R2 are equivalent. The coefficient of determination R2 is convenient because it is a dimensionless value usually in the range [0; 1], the value of R2 < 0 shows that the arithmetic mean x has a better result than the predictions of the constructed model). 

## Results

**Fig. 1.**
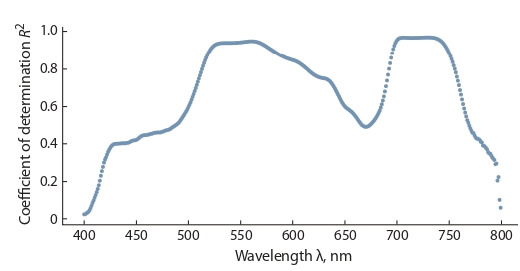
Determination coefficients of the obtained GGF models at λ [400; 800], which were calculated on the training sample.

Selection of parameters for the functional dependence method. For the GGM prediction on the training sample, we selected the coefficients α and β of equations (1), as well as the values λ so as to maximize the value of R2. The value λNIR = 800 nm is selected as the wavelength in the nearinfrared range. To get the coefficients α and β, we took a linear model based on the least squares method (the LinearRegression class from the sklearn.linear_model package). For each λ [400; 800] with a step of 1 nm, a specific type of GGM curve was found. The coefficients of determination R2 for the predictions of the obtained models are shown in Fig. 1. The highest coefficient of determination was achieved at the wavelength λ = 705 nm. The result is consistent with the recommended range λ [525; 555] [695; 725] (Gitelson et al., 2003). The RF method is compared with the GGM model obtained at this wavelength λ = 705 nm.

Results of constructing an algorithm based on the random forest method. The characteristics of the accuracy of the prediction of chlorophyll concentrations (the values of the mse, mae, R2 parameters) for all six models in the test sample are shown in the table. The RF-(400–800)-der and RF-(400–800)- der+der2 methods demonstrated high prediction accuracy. As the best of them, the RF-(400–800)-der method was selected as having a smaller number of input parameters.

**Table 1.**
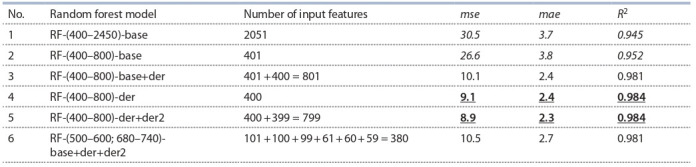
Results of a random forest model trained on different sets of input features Notе. The numbers in the description of the feature indicate the range of wavelengths. Additional characteristics of the features: base – reflection spectrum; der – values of the first derivative of the spectrum; der2 – values of the second derivative of the spectrum. The values with the worst accuracy are shown in italics, and the values with the best accuracy are highlighted in bold.

**Fig. 2.**
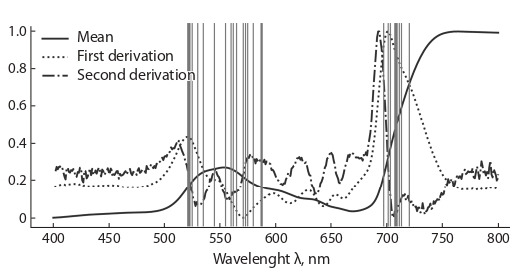
Characteristics of the reflection spectrum of sycamore maple pigment samples used for model training. The lines show: the average value of the intensity of the reflection spectrum Rλ (Y-axis) for different wavelengths (X-axis); the value of the first derivative of the average intensity; the value of the second derivative. The values of the derivatives are normalized to the interval [0; 1]. Vertical lines indicate the wavelengths whose spectrum intensities make the greatest contribution to the prediction accuracy of the RF-(400–2450)-base model.

The selection of wavelengths, the reflection coefficients for which were taken as input features for predicting chlorophyll concentrations by the random forest method, was carried out on the basis of the first model (RF-(400–2450)-base). This is due to the fact that at first it was not known whether the entire spectrum was needed, or only a part of it was necessary, and which one. As mentioned earlier, the RF algorithm allows you to evaluate the information content of the features the training took place on. After configuring the control parameters of the RF-(400–2450)-base model, we took the obtained parameters to re-train the models on five training samples (from crossvalidation). For these five models, we identified 10 features with the greatest contribution to the prediction. The results are shown in Fig. 2: the vertical lines represent the combined set of wavelengths, the spectrum intensities for which make the most significant contribution to the prediction accuracy (26 wavelengths out of 10∙5 = 50 possible if the values did not intersect). Interestingly, the most significant features lie in the visible range; most of these features are in the wavelength range of 500–600 and 680–740 nm. On this basis we have formulated the wavelengths of the input characteristics for the remaining five models for predictions by random forest (see above).

**Fig. 3.**
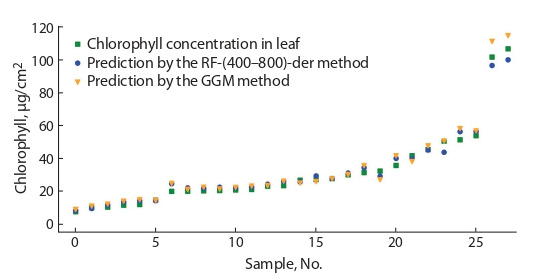
Comparison of true and predicted values of chlorophyll concentration in sycamore maple leaf tissues for validation sampling.

**Fig. 4.**
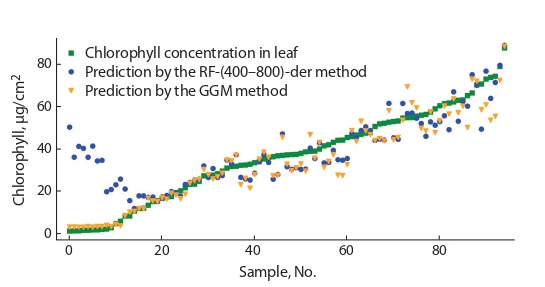
Comparison of true and predicted values of chlorophyll concentration in leaf tissues of not related to sycamore maple samples.

Comparison of the accuracy of the RF and GGM methods. The results of the comparison of the methods for predicting chlorophyll concentrations by the RF-(400–800)-der and GGM methods and their experimentally measured values at different concentrations are shown in Fig. 3 and 4. For sycamore maple samples (the type taken to fit the parameters), the RF-(400–800)-der method shows a better result compared to the GGM method: √mseRF = 3.01 µg/cm2 versus √mseGGM = 3.21 µg/cm2. When testing the methods on a sample of plant leaves from other species, the GGM functional dependence method has an advantage √mseGGM = 6.31 µg/cm2 versus √mseRF = 12.97 µg/cm2. The GGM method shows high accuracy at low concentrations of chlorophyll, while the RF method shows a large error at these values. However, in the range of chlorophyll concentrations above 20 µg/cm2, the RF-(400–800)-der algorithm has the best result: √mseRF = = 5.91 µg/cm2 versus √mseGGM = 7.01 µg/cm2

Further analysis revealed that for samples with a chlorophyll concentration of less than 7 µg/cm2, the reflection coefficients R550 (maximum of the reflection spectrum) and R680 (minimum of the reflection spectrum) are visually significantly different from all the others (Fig. 5, points in the upper right quarter). The predictions for these samples have a significant error. However, it was not possible to find out what the differences in the reflection spectrum are related to: these samples do not differ from the rest either in the surface density of the leaf or in the equivalent water thickness for the leaf (Jacquemound et al., 2003). Six out of ten plant species from these samples also have samples with normally predicted values. Further analysis of the causes of the anomalous spectrum is difficult, since the data are taken from open sources, and the measurements themselves were carried out more than 17 years ago.

## Discussion

Many studies on the application of reflection spectra to estimate pigment concentrations involve neural networks (Golhani et al., 2018), while the decisions founded on treebased methods are also common in machine learning research tasks. We used the decision tree method to predict chlorophyll concentrations in plant leaves and compared the results with the functional dependence method. We have found the ranges of the spectrum, the intensity of reflection in which most strongly affects the accuracy of the prediction by the random forest method. 

The range of 690–750 nm in the literature is called the red edge of photosynthesis (Curran et al., 1990; Gitelson et al., 2003; Croft, Chen, 2018), and the neighborhood of 550 nm, where the maximum of the chlorophyll reflection spectrum is located, is known as the green edge (Gitelson et al., 2003). As it can be seen from Fig. 2, in our study, these regions contain the most important predictors for the random forest method. The choice of a narrower wavelength range of the visible spectrum (400–800 nm) as input features compared to the full source data (400–2450 nm) improved the quality of the model. The explanation is that after dividing the sample into subspaces, some of them are less suitable for training, and the trees trained on these values introduce an error in the total result. The greatest effect was achieved with the use of derived spectral dependences.

The random forest RF method performed well when working with sycamore maple samples, while the functional dependence of GGM performed well when working with different plant species. This is due to the greater generalization ability of the GGM method, as it has fewer configurable parameters. However, the lower accuracy of the RF method on samples from other plant species is partly due to the small size of the training sample and the fact that only one species is represented in it. For example, the best results of the random forest method were achieved with a tree depth of 5 or 6, and this requires a minimum of 32 or 64 objects of the training sample, while the functional method (1) requires a minimum of two points (preferably a point at small values of chlorophyll and a point at large values). Apparently, this feature of the RF method can be eliminated by using more training data with samples from different plant species.

Nevertheless, the procedure for selecting parameters for the RF method showed that the most significant features for prediction lie in the visible region, but the influence of the plant structure was not taken into account in this method. Along with this, in the functional dependence (1), the structure of the plant tissue is taken into account by the RNIR member. If the experiment is performed with different plant species (see Fig. 4), then at low values of chlorophyll, the structure of the plant begins to play a significant role.

Interestingly, both methods work in the range λ [525; 555] [695; 725]. Both methods work on the decline of the derivative of the reflection spectrum, as it is shown in Fig. 2.

The word “random” in the name of the method “random forest” can lead to the idea that when you change the random parameter used by the algorithm, you can get radically different results. We believe that with reasonably selected control parameters, a reasonable division into training and test samples, this probability is low. In our case, 625 models were built for each set of input features (a search of a set of 125 combinations of control parameters, and 5 cross-checked models for each combination). In addition, it follows from the above table that the RF-(400–800)-base+der, RF-(400–800)- der, RF-(400–800)-der+der2 methods have similar results (and, importantly, have less mse compared to the GGM method), which indirectly confirms that the results will not change dramatically

## Conclusion

The random forest method is one of the algorithms for constructing functional dependencies using machine learning methods. Therefore, it can be used for mass automatic construction of functions that connect the observed features with the desired ones in monitoring tasks. The results of this work have shown that it is advisable to use the random forest algorithm (and similar ones) in the task of determining the content of chlorophyll in a plant leaf if there is a large sample, at least 32 elements, represented by a wide range of chlorophyll concentrations, while the structure of the leaf tissue changes slightly (for example, the application of the algorithm only on those plants on which it was trained). In other cases, it is better to give preference to methods based on empirical dependencies (such as the GGM method discussed here).

## Conflict of interest

The authors declare no conflict of interest.
